# Supplementary material for: Electrically controlled superconductor-to-failed insulator transition and giant anomalous Hall effect in kagome metal CsV3Sb5 nanoflakes
Source: Nat Commun. 2023 Feb 8;14:678. doi: 10.1038/s41467-023-36208-6 (PMC9908868; doi:10.1038/s41467-023-36208-6)
Supplement: Supplementary file 1 — Supplementary Information [file 41467_2023_36208_MOESM1_ESM.pdf]

Supplementary Information

Electrically controlled superconductor-to-failed insulator transition and giant anomalous Hall effect in kagome metal CsV_3Sb_5 nanoflakes

Guolin Zheng[1, 2#], Cheng Tan[1,3#], Zheng Chen[2#], Maoyuan Wang[4], Xiangde Zhu[2], Sultan Albarakati[1], Meri Algarni[1], James Partridge[5], Lawrence Farrar[1], Jianhui Zhou[2*], Wei Ning[2*], Mingliang Tian[2,6*], Michael S. Fuhrer[7], Lan Wang[1,3*]

[1]ARC Centre of Excellence in Future Low-Energy Electronics Technologies (FLEET), School of Science, RMIT University, Melbourne, VIC 3001, Australia.

[2]Anhui Province Key Laboratory of Condensed Matter Physics at Extreme Conditions, High Magnetic Field Laboratory, HFIPS, Chinese Academy of Sciences (CAS), Hefei 230031, Anhui, China.

[3]Department of Physics, and Lab of 2D Materials and Quantum Devices, School of Physics, Hefei University of Technology, Hefei, Anhui 230009, China.

[4]International Center for Quantum Materials, School of Physics, Peking University, Beijing 100871, China.

[5]School of Science, RMIT University, Melbourne, VIC 3001, Australia.

[6]School of Physics and Optoelectronic Engineering, Anhui University, Hefei 230601, Anhui, China.

[7]ARC Centre of Excellence in Future Low-Energy Electronics Technologies (FLEET), Monash University, Melbourne, VIC 3800, Australia.

[#] These authors equally contributed to the paper.

[*] Corresponding authors. Correspondence and requests for materials should be addressed to J. Z. (jhzhou@hmfl.ac.cn), W. N. (email: ningwei@hmfl.ac.cn), M. T. (tianml@hmfl.ac.cn) and L. W. (email: wanglan@hfut.edu.cn).

Supplementary section 1: Synthesis and basic characterization of bulk CsV_3Sb_5 crystals

Bulk CsV_3Sb_5 single crystals used in our experiments were synthesized via Sb flux method. The elemental Cs, V and Sb were mixed at a molar ratio of 1:3:20, and loaded in into a MgO crucible. This process was performed in a glove box in Ar ambience. Then the crucible was sealed in a vacuumed quartz tube. The ampule was slowly heated to 1000 ˚C and kept for 20 hours. After cooling at a rate of 2 ˚C/min, the extra flux was removed by fast centrifuging at 640 ˚C. Supplementary Fig. 1 shows the basic characterization of as-grown bulk CsV_3Sb_5 single crystals, including the X-ray diffraction pattern and the elemental ratio detected by the energy dispersive spectrometer.

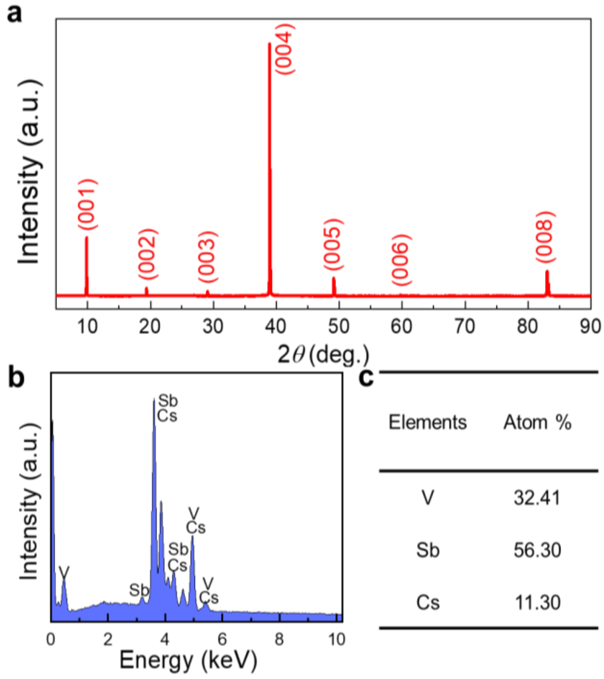

c

Elements	Atom %
V	32.41
Sb	56.30
Cs	11.30

Supplementary Fig. 1. Characterization of bulk CsV_3Sb_5. a X-ray diffraction pattern of CsV_3Sb_5 with (00l) diffraction peaks. **b** The energy dispersive spectrum and **c** the elemental ratio of bulk CsV_3Sb_5.

Supplementary section 2: Transport properties of CsV_3Sb_5 crystals

CsV_3Sb_5 single crystal exhibits CDW transition at 90 K, the superconducting transition at 2.5 K and Shubnikov-De Hass (SdH) oscillations under high fields at 2 K, as shown in Supplementary Fig. 3**a** and 3**b**. Anomalous Hall effect within ± 2 T was also observed in bulk CsV_3Sb_5 crystals. Supplementary Fig. 3**c** and 3**d** show the Hall resistivities of bulk CsV_3Sb_5 at various temperatures and the anomalous Hall resistivities (AHE) extracted by subtracting the linear Hall background at high fields, respectively. The anomalous Hall conductivity (AHC) was obtained by inverting the resistivity matrix, $\sigma_{xy}^{AHC} = -\rho_{yx}/\left(\rho_{xx}^2 + \rho_{yx}^2\right)$, as shown in the inset of Supplementary Fig. 2**d**. Recent spectroscopy experiments revealed that the Fermi level of pristine CsV_3Sb_5 lies slightly above (van Hove singularity) VHS1 near the M points [1]. The observed superconductivity, CDW as well as giant AHE in our bulk CsV_3Sb_5 are qualitatively in line with those reported single crystals [2]. These similarities revealed that our crystal should share the same Fermi level with those reported high quality CsV_3Sb_5 crystals. In our exfoliated nanoflakes, however, the Fermi level would be shifted slightly downward, due to the Cs vacancies [3-5], as discussed in the main text.

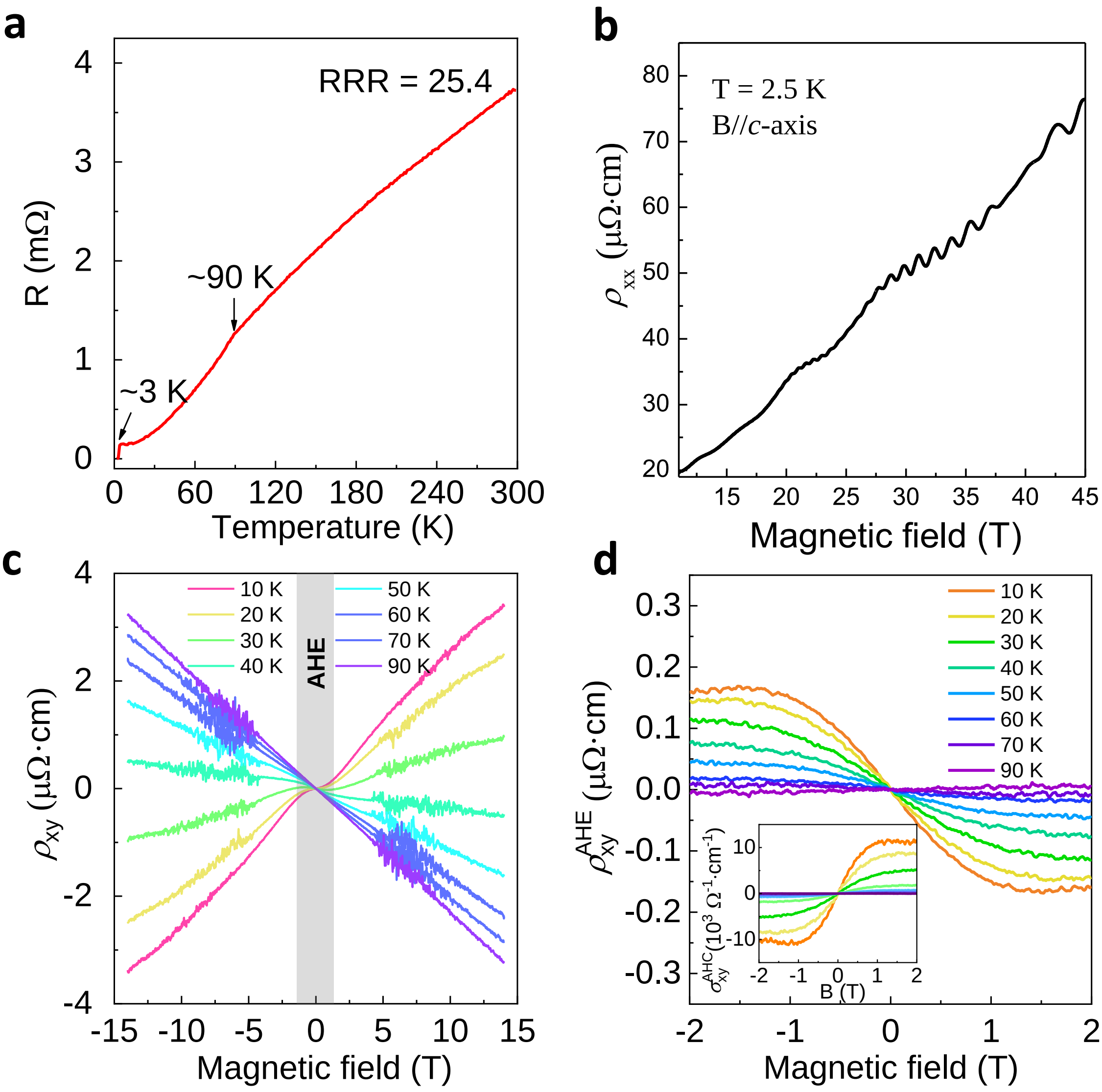

Supplementary Fig. 2. **Transport properties of bulk CsV_3Sb_5 crystals. a** The longitudinal resistance as function of temperature in CsV_3Sb_5 single crystal. **b** The high-field longitudinal magnetoresistivity of CsV_3Sb_5 at 2 K, which exhibits a prominent sdH oscillation at high field region. **c** Temperature dependent Hall resistivities under various temperatures. **d** The corresponding AHE obtained by linearly subtracting the ordinal Hall component at high fields. The inset shows the AHC components after inverting the resistivity matrix.

Supplementary section 3: Doping-induced quantum phase transition in CsV_3Sb_5 with various thicknesses

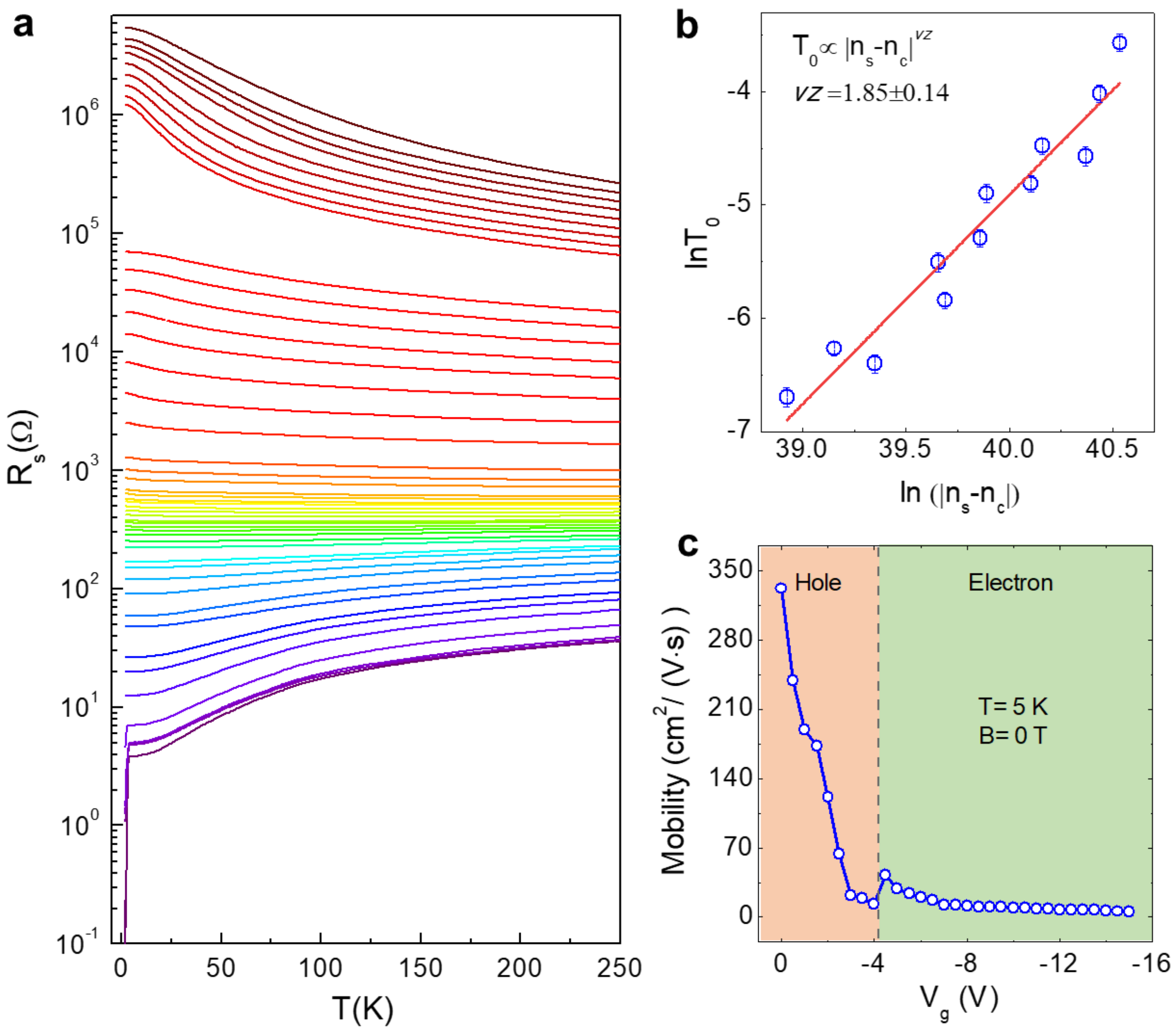

Supplementary Fig. 3. Detailed measurements for device #5 under various gating voltages. **a** Shows the detailed $R_s(T)$ curves under different gate voltages in device #5 (with thickness 21 nm). For the metallic state ($n_s > n_c \sim 7.91 \times 10^{17} m^{-2}$), $\rho(T) \propto exp\ (T/T_0)^{1/2}$ was utilized to fit the $R_S(T)$ curves within 2~50 K. While for the insulating state ($n_s < n_c$), we used $\rho(T) \propto exp\ (T_0/T)^{1/2}$ to fit the $R_S(T)$ curves within 2~50 K. **b** Scaling parameter T_0 as a function of $|n_s - n_c|$ for device #5. The solid red line is the linear fitting with $\nu z = 1.85 \pm 0.14$. **c** Gate-dependent mobility at T=5 K, B=0 T. The mobilities of electrons and holes exhibit distinct behavior against the protonic gate.

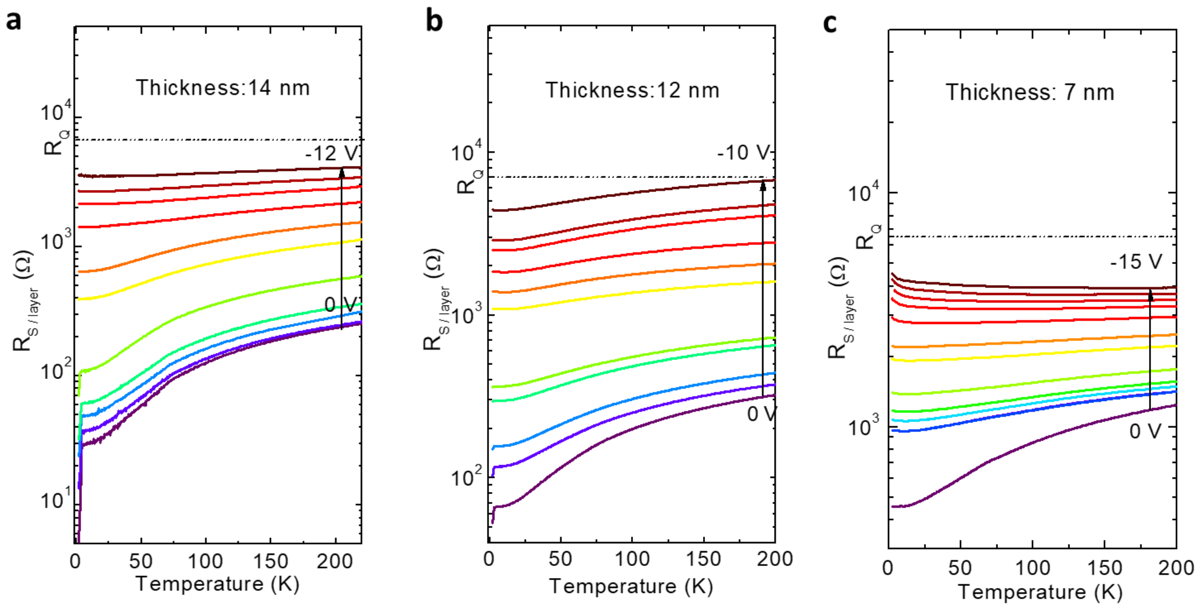

Supplementary Fig. 4. Temperature-dependent sheet resistance per layer in thinner CsV_3Sb_5 devices under various gating voltages. a-c Temperature dependent sheet resistance per layer under different gate voltages for 14-7 nm devices, respectively. The CDW transitions gradually disappear with the increasing intercalated protons. As discussed in main text, superconductor-to-failed insulator transition occurs at a critical sheet resistance R_c. If we convert the critical sheet resistance R_c to the sheet resistance per layer, we can find that this critical sheet resistance per layer is very close to the quantum resistance R_Q in device #5 and #8. In these three samples ranging from 14-7 nm, however, the sheet resistance per layer did not reach the quantum resistance R_Q due to the failure of those devices under high gate voltages, demonstrating a superconductor-to-metal transition. The dashed line is the guideline of the quantum resistance R_Q.

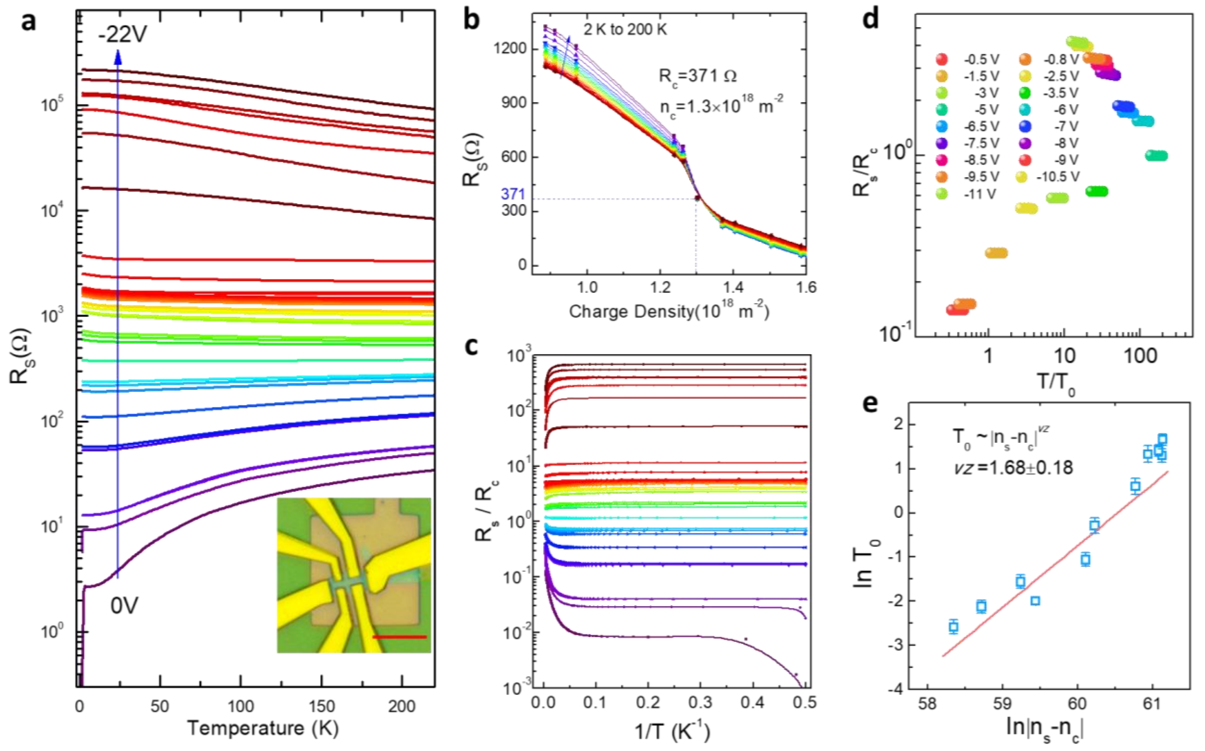

Supplementary Fig. 5. Superconductor-to-failed insulator transition in device #8 (18 nm) under various gating voltages. a. Gate-dependent sheet resistance. Akin to device #5, we observed a superconductor-to-failed insulator transition with a critical sheet resistance $R_c \approx 371\ \Omega$, as shown in **b**. If we convert the critical sheet resistance to the sheet resistance per layer, it has a value of $7420\ \Omega$, close to the quantum resistance of Cooper pair (R_Q). **c** Shows the sheet resistance as the function of 1/T under different gate voltages. Akin to device #5, it exhibits a 'failed insulator' state with a saturated resistance for T → 0 K on insulating side. **d** Multiple sets of $R_s(T, B)$ curves can collapse onto a single function, akin to a 2D SIT. **e**. By extracting the exponent product νz and plotting lnT_0 versus $ln|n_s - n_c|$ curve, we get $\nu z = 1.68$, close to the value obtained in sample #5.

Supplementary section 4: Preparation of protonic gate and its tunability on charge density wave (CDW) and superconductivity (SC) in thick CsV_3Sb_5 flakes

To prepare solid protonic electrolyte, we first mixed tetraethyl orthosilicate (from Alfa Aesar), ethanol, deionized water, phosphoric acid (as a proton source, from Alfa Aesar, 85% wt%) with a typical molar ratio 1:18:6:0.03, then the mixed solution was stirred for 2 hours and annealed for another 2 hours at 50 ℃ in a sealed bottle to form polymerized Si–O–Si chains. Finally, the substrate with bottom gate electrodes was spin-coated with the prepared protonic solution and baked at 150 ℃ for 25 mins.

In the relatively thick flakes (around 100 nm) with carrier density around $2 \times 10^{22}\ cm^{-3}$, we found that the tunability of SC and CDW is very limited as shown in Supplementary Fig. 6. Under various gate voltages, we found that the onset SC transition temperature T_c^{onset} is between 4.5 K and 4.9 K while the offset SC transition temperature T_c^{offset} is around 3 K for gate voltages 4.5 V $\leq V_g \leq$ 5.8 V. For other gate voltages, the sheet resistance does not approach zero before 2 K, as shown in Supplementary Fig. 2c. Both the onset and offset SC transition temperatures are insensitive to the gate voltages, revealing the robustness of SC phase against the moderate charge doping. Supplementary Fig. 6d shows the derivatives of the RT curves at different gate voltages. The CDW transition temperature T_{cdw} of around 72 K remains almost unchanged between $V_g = -6.1$ Vand 4.5 V but increases to 79 K when the V_g exceeds 5.3 V.

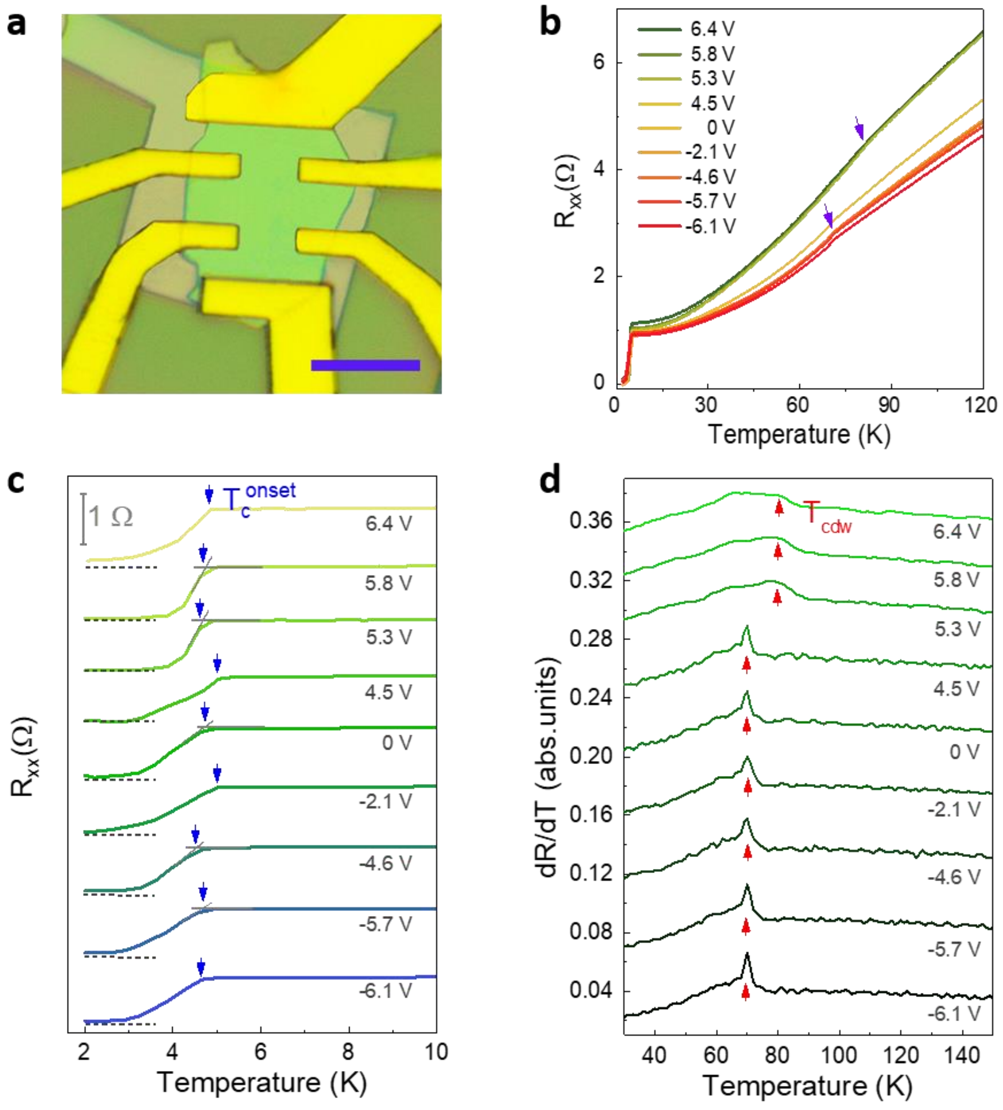

Supplementary Fig. 6. Temperature-dependent R_{xx} curves under various gating voltages in device #4. a Optical image of device #4 on a solid proton conductor. The scale bar represents 10 μm. **b** Temperature-dependent longitudinal resistance $R_{xx}(T)$ under different gating voltages in device #4. **c** The superconducting transition temperature T_C^{onset} at various temperatures. The dashed lines are the zero-resistance lines for each curve. **d** Shows the derivatives of the resistance curves $R_{xx}(T)$ under different voltages. As the voltage changes from -6.1 V to 6.4 V, the CDW transition temperature T_{cdw} abruptly increases from 70 K to 79 K at $V_g = 5.3\ V$.

Supplementary section 5: Extended Hall effects under other gating voltages in device #4

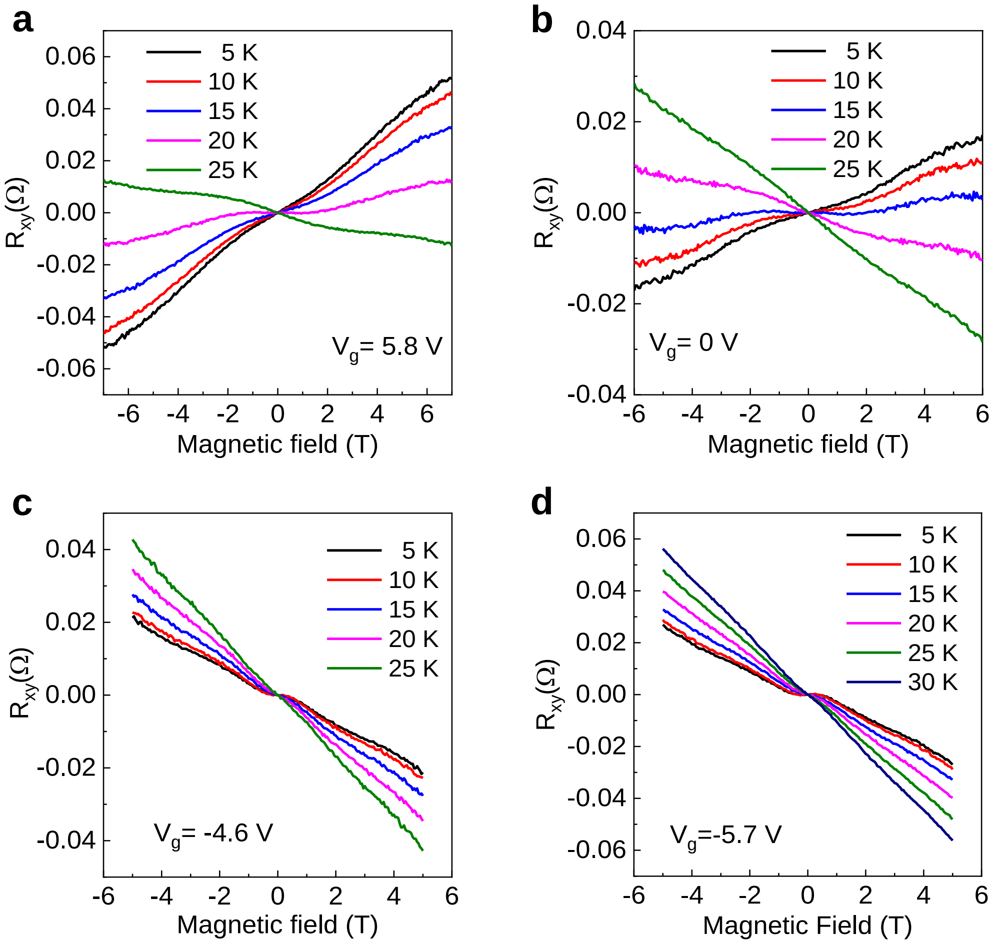

Supplementary Fig. 7. Temperature-dependent Hall effects in device #4 under other different gating voltages. The Hall effects exhibit a sign reversal at $V_g < -2.7$ V, where the transport is mainly dominated by the electron pockets. Similar sign change in RbV_3Sb_5 was probably attributed to the enhanced mobility of hole-type carriers [6]. Moreover, the Hall effects under different temperatures exhibit no sign reversal in electron pockets, demonstrating the disappearance of the temperature-induced band renormalization in these electron pockets.

Supplementary section 6: Gate dependent Hall effects in device #4 at 5 K

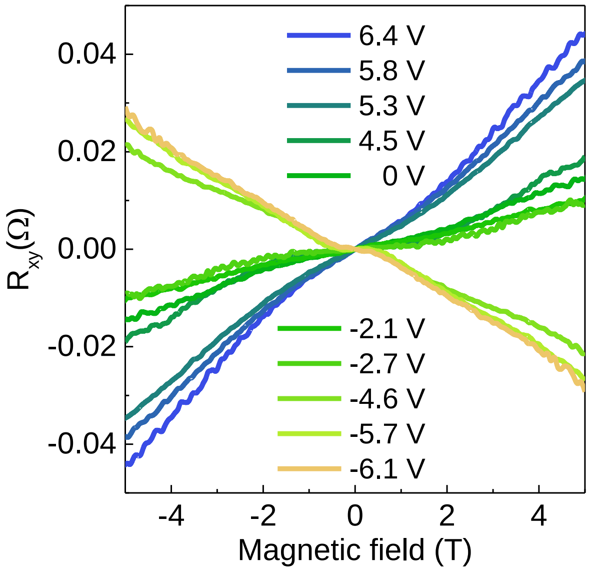

Supplementary Fig. 8. Gate dependent Hall effects in device #4 at 5 K. Hall effects exhibit a positive slope (hole type carriers) at high fields with $-2.7\,V \leq V_g \leq 6.4\,V$. However, the Hall slope changes to negative (electron type) when $V_g < -2.7\,V$, demonstrating a dramatic change of Fermi surface topology. Akin to Supplementary Fig. 2, we can subtract the ordinary Hall components ρ_{xy}^N and get the anomalous Hall components by linearly fitting the Hall resistivities at high fields.

Supplementary section 7: Hall effects in some other gated samples

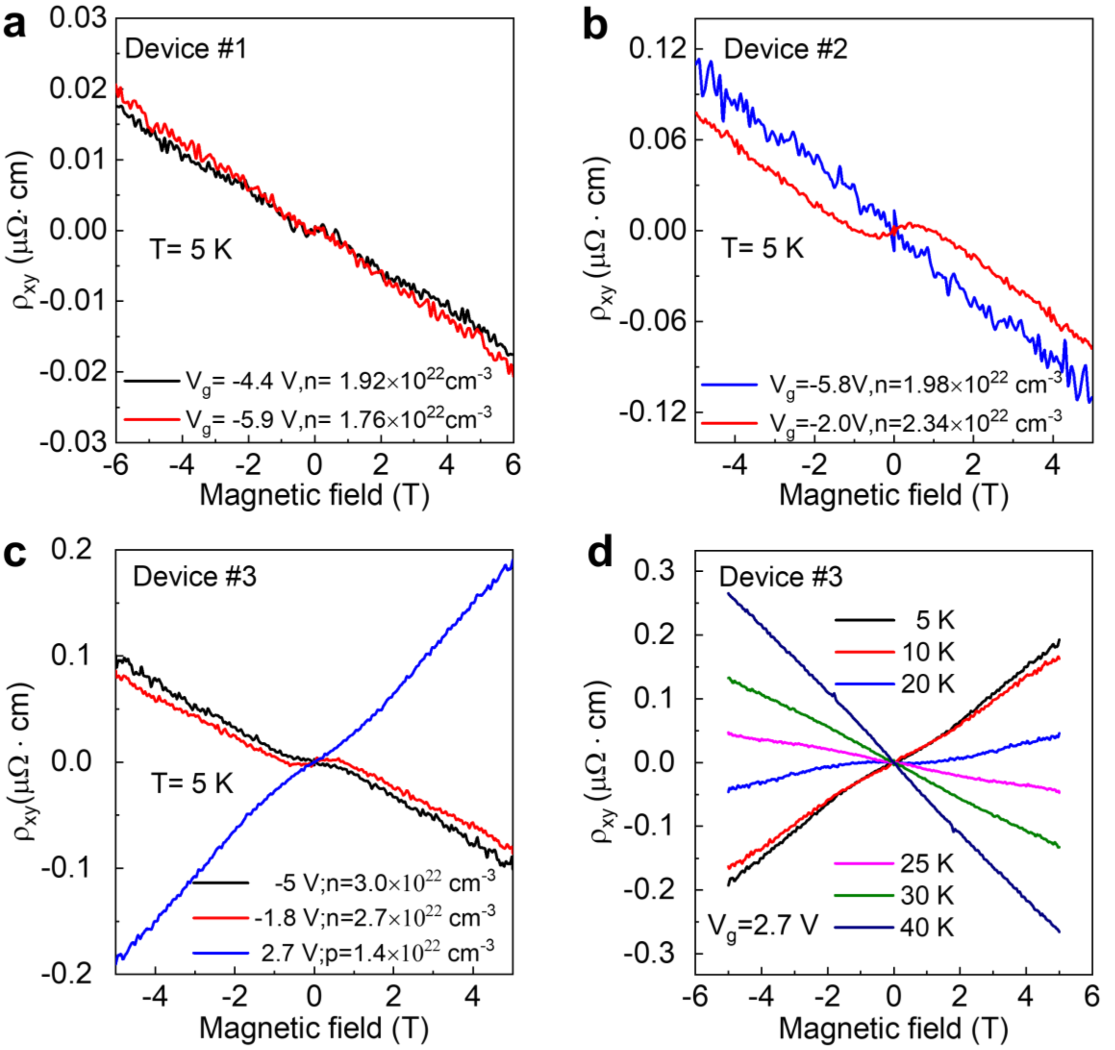

Supplementary Fig. 9. Gate dependent Hall effects in devices #1, #2 and #3. a Gate dependent Hall effects in device #1 (with thickness 90 nm) at T = 5 K. Due to the linear Hall resistivity at low field regions, device #1 shows no anomalous Hall effects under these gate voltages. **b** Hall effects of device #2 (with thickness 85 nm) at T = 5 K with $V_g = -2$ V and -5.8 V. The observed anomalous Hall effect at -2.0 V disappeared at $V_g = -5.8$ V. **c** Hall effects of device #3 (with thickness 95 nm) at 5 K under various gate voltages. The Hall exhibits a sign reversal when sweeping the gate voltage from 2.7 V to -1.8 V, akin to the device #4. **d** The Hall effects of device #3 at $V_g = 2.7$ V at various temperatures.

Supplementary section 8: σ_{xy}^{A} versus σ_{xx} for different materials

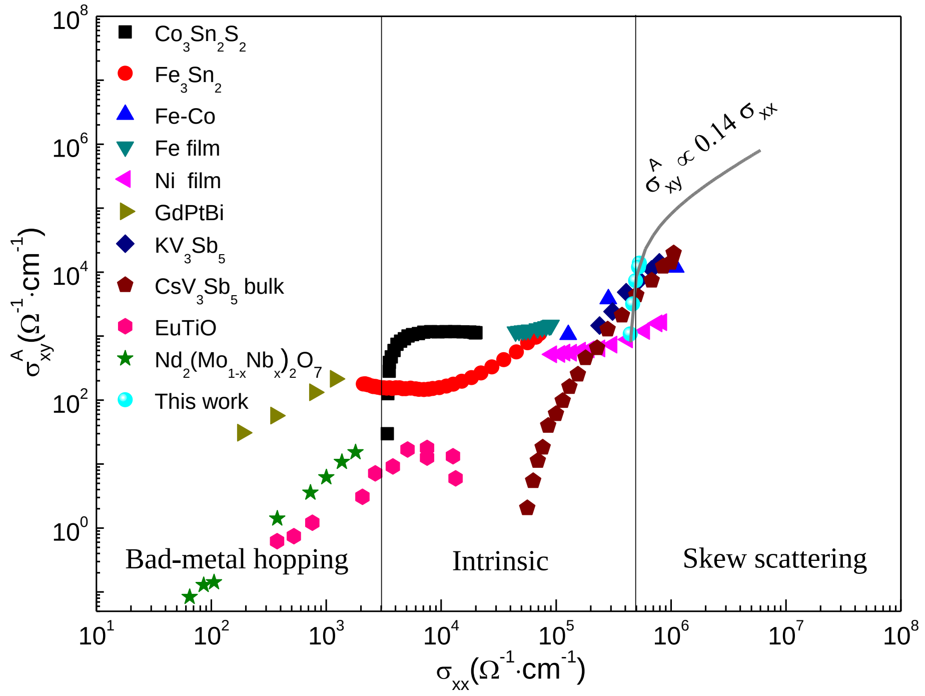

Supplementary Fig. 10. σ_{xy}^{A} versus σ_{xx} for a variety of materials. There are three different regimes including bad-metal hopping, intrinsic and skew scattering spaced by vertical lines. As we can see, the data in this work locates at the same region with previous studied KV_3Sb_5 and CsV_3Sb_5. However, different from previous studies where $\sigma_{xy}^{A} \propto \sigma_{xx}{}^{2}$, the maximal AHC obtained in proton intercalated devices exhibit a linear scaling relation $\sigma_{xy}^{A} \propto 0.14\ \sigma_{xx}$. Data from ref. [2,7-16].

Supplementary section 9: The contribution of skew scattering and side jump to AHE in CsV_3Sb_5

9-1. Skew scattering.

1. scenarios for the skew scattering--In this section, we would like to carefully clarify the skew scattering's contribution to the giant AHE in CsV_3Sb_5. It has been known that the extrinsic skew scattering of AHE essentially originates from the asymmetric scattering of electrons by nonmagnetic/magnetic impurities. Generally speaking, there are three main scenarios to produce the extrinsic skew scattering and the resultant AHE (Sec. IV B in [16]). First, in ferromagnetic (FM) metals, the spin-orbit coupling leads to the asymmetric scattering of a carrier from impurities and introduces a momentum perpendicular to both incident momentum and the magnetization. This scenario had been detailedly examined in 2D FM Rashba model [17] and 2D massive Dirac fermions [18] with nonzero Berry curvature and finite AHE. Both the scalar and magnetic impurities could induce the extrinsic AHE including the skew scattering and side jump contributions. The linear scaling law between the anomalous Hall conductivity and the longitudinal one was derived [17]. Note that the finite temperature could enhance the scattering rate of carriers against phonons and decrease the mobility and the longitudinal conductivity. As a result, the extrinsic AHE would diminish. Second, the Kondo theory for skew scattering depends on the spin waves of local moments at finite temperature that lead to asymmetric scattering. Recently, this scenario was generalized to the case with spin chirality, accounting for the AHE in chiral magnets. Moreover, it has been also proposed to explain the appearance of giant AHE in kagome metals AV_3Sb_5. However, the nearly vanishing of local magnetic moments of V atoms and the resulting spin wave excitations may rule out the Kondo theory for the giant AHE in AV_3Sb_5. Third, the resonant skew scattering from the scattering of carriers from the virtual bound states in magnetic ions dissolved in nonmagnetic

metallic systems (such as heavy-fermion systems). The resonant skew scattering scenario requires both the spin orbital coupling (SOC) and the local moments of magnetic d- or f-electrons. However, the lack of typical temperature-dependence of anomalous Hall resistivity due to the resonant skew scattering and the nearly vanishing of local magnetic moments could rule out the resonant skew scattering scenario for the giant AHE in AV_3Sb_5.

The giant AHE in AV_3Sb_5 can be attributed to the intense extrinsic skew scattering of holes in the nearly flat bands of VHS1 at the saddle points by multiple impurities. We now estimate the concentration of impurities in our CsV_3Sb_5 samples from some empirical formulas for the skewness factor $S \approx \frac{E_{SO}}{E_F} \cdot (\tau n_{imp} n_{imp} V_{imp})$, where τ is the relaxation time, h is the Planck's constant, the spin-orbital interaction energy ESO refers the CDW gap for chiral CDW phase, E_F is the Fermi energy, $n_{imp}$is the impurity concentration and V_{imp} is the impurity potential strength [17]. The approximate concentration of impurities to be the order of 1%, which includes the nonmagnetic impurities and paramagnetic impurities.

2. analysis of previous results--Let us turn to discuss the recent experimental results of AHE in the kagome family compounds AV_3Sb_5. In Ref. [14] for the AHE in KV_3Sb_5, the AHE was attributed to the scattering of Dirac quasiparticles by chiral spin clusters of V atoms. The authors proposed a quadratic scaling between anomalous Hall conductivity and the longitudinal one, which is distinct from the linear scaling law for the extrinsic skew scattering mechanism for AHE. However, recent spin-muon scattering did find no evidence of sufficiently large local moment of V atom, thus the mechanism was ruled out. Moreover, Ref. [14] neither identified the existence of CDW order nor established the connection between CDW and AHE. In Ref. [2], similar AHE was observed in CsV_3Sb_5 and was proposed to obey the quadratic scaling law as KV_3Sb_5 in Ref.[14]. The evolution of AHE under high pressure further revealed the possible connection between AHE and CDW order. The authors instead suggest that the giant AHE may

come from enhanced skew scattering in the CDW state and large Berry curvature due to the kagome lattice. Recent ARPES experiments revealed the multiple energy scales for CDW order: 70 meV for a band forming a saddle point near the M points and the smaller gap (~18 meV) for a band forming Dirac cones [19]. However, the transport experiment in Ref.[2] did not unambiguously determine which energy bands for CDW phase would dominate the giant AHE. That is, whether the Dirac fermions, the heavy fermions near the saddle points play a crucial role in the AHE? Meanwhile, the authors did not clarify the role of the electron bands near the G-point in the AHE. More importantly, the authors did not determine which scenario would dominate the skew scattering contribution to giant AHE, which is crucial to reach a microscopic model to quantitatively understand the giant AHE. Due to limitation of transport data from samples with fixed carrier density, there is no definitive magnitude of intrinsic AHE entirely from the Berry curvature of energy bands. In addition, some controversy between the well-known linear scaling law for anomalous Hall conductivity and the longitudinal one [17] and the quadratic scaling law proposed in Refs.[2] and [14] need to be clarified.

3. our results--First-principles calculations and spectroscopy experiments reveal three distinct Fermi surface pockets in all three kagome compounds AV_3Sb_5: (i) a circular pocket around the G-point that originates from the electronic states in the pz orbital of Sb (ii) Dirac cones with dominant d_{xy}, $d_{x^2-y^2}$ and d_{z^2} character (iii) heavy-hole bands of d_{xz} and d_{yz} orbitals of V atoms near the saddle points [20].

3.1 electrons near the G point--We first briefly consider the impacts of the electron bands near the G-point. These topologically trivial bands do not support Berry curvature and thus not contribute to the intrinsic AHE. Moreover, none of these energy bands near the G-point have contribution to the extrinsic AHE primarily due to their irrelevance to the CDW transition.

3.2 Dirac bands--Let us consider the contribution of AHE from the Dirac fermions in AV_3Sb_5. The intrinsic AHE is proportional to the distance in momentum space between the Weyl nodes with opposite chirality. For the Dirac semimetals, the time reversal symmetry forces the intrinsic AHE to vanish identically. Applied an external magnetic field, the Dirac nodes are split into a pair of Weyl nodes, resulting in a finite AHE. It has been shown that the giant AHE in AV_3Sb_5 appears when the magnetic field is as weak as 2 Tesla. The magnetic field could induce splitting of Weyl nodes for typical Fermi velocity (V_F =10^5 m/s) and g-factor (g =2) (the Zeeman splitting is typically ~0.23 meV and the corresponding distance of Weyl nodes with opposite chirality in momentum space is about $3.5 \times 10^6\ \mathrm{m}^{-1}$), and the resultant AHE is about $1.3\ \Omega^{-1}\mathrm{cm}^{-1}$, which is far smaller than the intrinsic AHE observed in recent experiments in AV_3Sb_5. Since this intrinsic contribution from Dirac bands is not much sensitive to temperature within the CDW transition temperature, the nearly vanishing AHE at slightly high temperatures observed in recent experiments (such as refs. [23-25] in the main text) implies that the real intrinsic AHE from Dirac bands is negligible. Recent numerical study shows that the extrinsic AHE is nearly independent of the longitudinal one and significantly smaller than the intrinsic one in the doped Weyl semimetals or Weyl metals [21]. It is inconsistent with the fact that the extrinsic AHE dominates over the intrinsic one in AV_3Sb_5. As a result, the total AHE including intrinsic and extrinsic parts from Dirac bands could not mainly account for the giant AHE in the kagome family compounds. It is worth noting that the particle-hole symmetry of the Dirac type quasiparticles implies a symmetric magnitude of AHE in both the hole and electron pockets. However, the significant difference in the evolution (both magnitude and trend) of the AHE in both electron and hole pockets in our experiments suggests that Dirac type quasiparticles with particle-hole symmetry unlikely leads to the giant AHE. Therefore, the giant AHE was mainly contributed by the heavy-hole bands with nonzero Berry curvature near the saddle points in the Brillouin zone.

3.3 bands near the saddle points--In our experiments, by use of a protonic gate, we found that the carrier density and the corresponding Fermi surface topology could be significantly modulated in CsV_3Sb_5 nanoflakes. Meanwhile, shifting the Fermi level across the CDW gap, we successively observed a giant AHE (~$15000\ \Omega^{-1}cm^{-1}$, mainly from the hole pocket), a moderately large AHE (~$1100\ \Omega^{-1}cm^{-1}$, near the electron-hole crossover point), and a large AHE (several thousands of $\Omega^{-1}cm^{-1}$, mainly from the electron pocket), in line with the asymmetric DOS around the VHS1 due to the opening of CDW gap [1]. It is qualitatively consistent with the fact that in the high conducting regime, the skew scattering conductivity of the prototypical model for AHE is usually proportional to the scattering time and the density of states near the Fermi level [17]. Scaling relations σ_{xy}^{A} vs σ_{xx} under various gate voltages first validated a clear extrinsic-to-intrinsic transition of AHE and unveiled an intrinsic AHC due to the chiral charge order. We extract the linear scaling law of σ_{xy}^{A} vs σ_{xx} against the gate voltages, which is compatible with the known empirical scaling relation and previous theoretical prediction. The giant AHE in AV_3Sb_5 is dominated by the intense extrinsic skew scattering of holes in the nearly flat bands of VHS1 with finite Berry curvature due to the Vanadium vacancies and FM fluctuation of paramagnetic impurities, similar to the way for 2D FM Rashba model [17] and 2D massive Dirac fermions [18], in which the extrinsic skew scattering can be dominant in AHE in the high conducting regime. Thus, our experimental findings establish a global picture of the AHE against the Fermi energy and further unveil its correlation with the possible chiral charge order in the topological kagome lattice AV_3Sb_5.

9-2. Side jump.

In this section, we would like to briefly clarify the side jump contribution to AHE, which is inevitable due to the disorder scattering. The side-jump mechanism could be viewed as a consequence of the anomalous velocity mechanism due to the Berry curvature acting while a

quasiparticle was under the influence of the electric field due to an impurity [16]. The ultra-high longitudinal conductivity in AV_3Sb_5 indicates the low concentration of impurities, implying that the side jump contribution is probably smaller than the intrinsic one. According to the empirical scaling law between the anomalous Hall conductivity and the longitudinal one [17], the side jump contribution is usually independent of the scattering time contributed from the intrinsic Berry phase. As shown in Figure 4a in the main text, the contribution that is independent of the longitudinal conductivity is about $1100\ \Omega^{-1}\mathrm{cm}^{-1}$, only 7% of the giant AHE observed in AV_3Sb_5. Thus, we conclude that the side jump mechanism made a minor contribution to the giant AHE. Note that these two contributions vary distinctly with respect to the material-dependent parameters such as the Fermi level, the exchange splitting and the strength of spin-orbital coupling.

Supplementary section 10: Theoretical calculations

The first-principles calculations are performed using Vienna ab initio simulation package (VASP) [22, 23] based on the density function theory with Perdew-Burke-Ernzerhof (PBE) parameterization of generalized gradient approximation (GGA) [24]. The energy cutoff of the plane wave basis is set as 300 eV, and the Brillouin zone of CsV_3Sb_5 is sampled by 12 × 12 × 6 k-mesh. The U value of d orbitals for V atom is set as 2 eV. The calculation of density of states and the intrinsic anomalous hall conductivity are calculated based on the Hamiltonian of maximally localized Wannier functions (MLWF) [25] with d orbitals of Vanadium atoms and p orbitals of Sb atoms, sampled by k-mesh of 160 × 160 × 100 with an adaptive refinement mesh of size of 3 × 3 × 3. The spin-orbit coupling was included in all numerical calculations.

Supplementary section 11: Band structure, the density of states for nonmagnetic CsV_3Sb_5

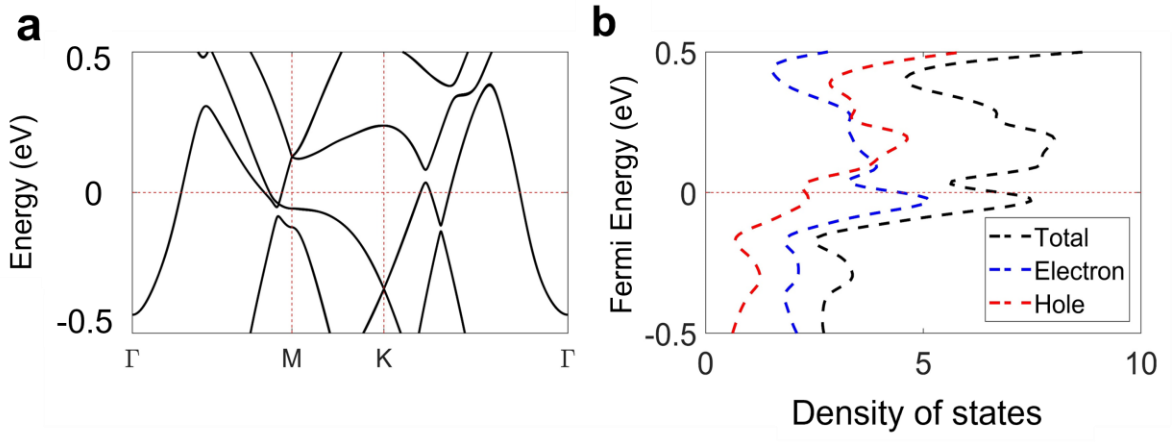

Supplementary Fig. 11. Band structure and the density of states in nonmagnetic CsV_3Sb_5. **a** The energy bands along the Γ-M-K-Γ direction for nonmagnetic CsV_3Sb_5. **b** The corresponding density of states for electrons with positive effective mass (blue), holes with negative effective mass (red) and the total one (black) in nonmagnetic phase.

Supplementary section 12: Band structure, the density of states and intrinsic anomalous Hall conductivity in ferromagnetic CsV_3Sb_5

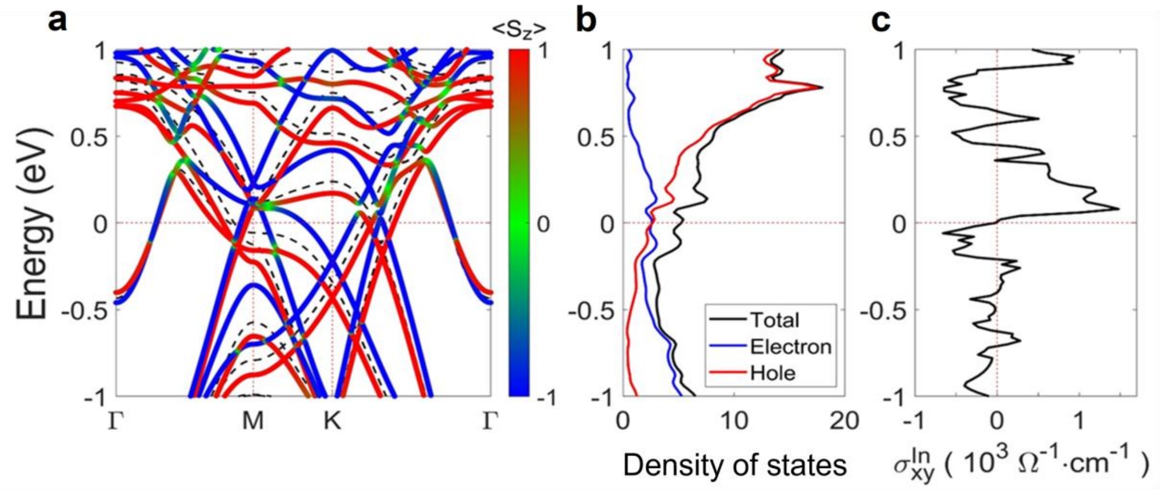

Supplementary Fig. 12. Band structure, the density of states and intrinsic anomalous Hall conductivity in CsV_3Sb_5. **a** The energy bands along the Γ-M-K-Γ direction for nonmagnetic (dashed lines) and ferromagnetic (colored solid lines) CsV_3Sb_5 **b** The corresponding density of states (DOS) for electron-type carriers (blue) and hole-type carriers (red) and the total DOS (black). The electrons and holes coexist and compete in a broad energy region around the Fermi energy. **c** The distribution of intrinsic anomalous Hall conductivity due to the Berry curvature of Bloch bands. The ground-state magnetic moment of each Vanadium atom was set 0.25 μ_B. Note that the realistic intrinsic anomalous Hall conductivity is much smaller than this calculated one due to the tiny magnetic moments of Vanadium atoms [26].

Supplementary section 13: Distributions of the orbital components of the energy bands for CsV_3Sb_5

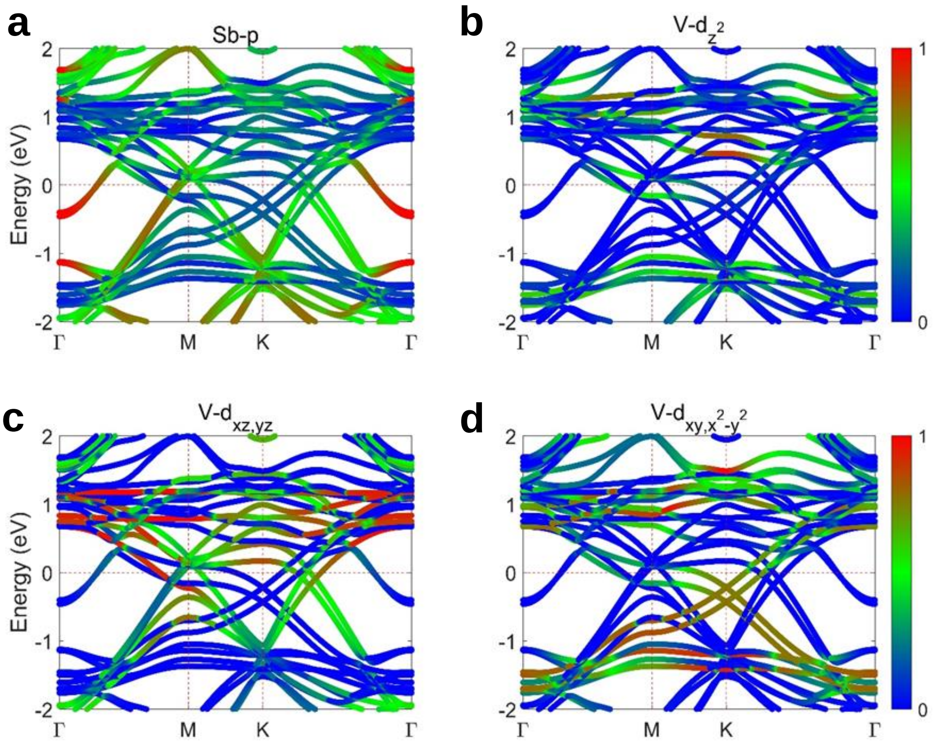

Supplementary Fig. 13. The energy bands along the Γ-M-K-Γ direction for ferromagnetic CsV_3Sb_5 and the distributions of the orbital components of the p-orbital for Sb atoms and the d-orbitals of V atoms. a The p-orbitals of Sb atoms mainly contribute to free-electron-gas-like bands around Γ point in the Brillouin zone. **b-d** The distribution of five d-orbitals of V atoms in the energy bands. **b** The d_z^2 orbital contributes slightly around the Fermi energy, due to its coupling with p-orbitals of Sb atoms. **c** Both the $d_{xz,yz}$ orbitals contribute mainly to a bunch of nearly flat bands around 1 eV, resulting in a large density of states. **d** The d_{xy,x^2-y^2} orbitals contribute to the flat bands around 1 eV and those around -1.5 eV. The four d-orbitals ($\mathrm{d}_{xz,yz}$ and d_{xy,x^2-y^2}) dominate the Dirac cones at K points as well as the van Hove singularities near the Fermi energy [27]. Note that these van Hove singularities at the M points are expected to play a key role in charge density order [28] and other correlation effects [20, 29, 30].

Supplementary section 14: The evolution of AHE against the gate voltage in device #4

According to the calculated band structure in Fig. 4b in main text, the conventional electrons near the Γ point remain almost unchanged during the CDW transition and thus do not significantly contribute to the AHE. In exfoliated nanoflakes, however, the increasing Cs vacancies would significantly lower the Fermi level downward (black arrow) due to the hole doping. Accordingly, the AHC at $V_g = 0\ V$ reduces to about one third of the maximal value, i.e., $4500\ \Omega^{-1}cm^{-1}$.

Applying a negative gate voltage with $V_g < -2.7\ V$, the Hall exhibits a sign reversal and the electrical transport is dominated by the electron-type carriers. According to the band structure in Supplementary Fig. 12, the DOS is dominated by the hole (electron) type carriers when the Fermi level roughly lies above (below) the VHS1. The electron-type carriers revealed at $V_g < -2.7\ V$ indicate that the negative voltages will further lower the Fermi level (similar to the hole doping). This is also in line with the broad competition region between electrons and holes in the calculated DOS in Supplementary Fig. 12. The AHC tends to vanish in the electron pocket in devices #1 and #2 when $n < 2 \times 10^{22}\ cm^{-3}$ [Fig. 3(c) and Supplementary Fig. 9], the corresponding Fermi level should go out of the lower CDW sub-band. Those observations demonstrate that the AHE in AV_3Sb_5 strongly depends on the relative position of the Fermi level with respect to the VHS1. Generally, the extrinsic contributions to the AHE require intense scatterings between the impurities (magnetic/nonmagnetic) and substantially mobile carriers near the Fermi level [16]. The low-temperature magnetic susceptibility exhibits a Curie tail below T_{cdw}, indicating the existence of sizeable amount of paramagnetic (PM) or superparamagnetic impurities [2], acting as a source of the scattering centers. The observed giant (around $V_g = 4.5$ V in the hole pocket), intrinsic (at $V_g = -4.6$ V with Fermi level being

in the middle of CDW gap) and large (around $V_g = -5.7$ V in the electron pocket) AHC are closely associated with the dramatic change of DOS while shifting Fermi level across the CDW gap. When the Fermi level approaches the upper (hole pocket) or lower (electron pocket) sub-band, the strength of extrinsic scattering will be greatly enhanced by the large DOS due to the flatness of bands, giving rise to the large AHE. Note that the distributions of DOS in the upper and lower sub-bands are clearly asymmetric with respect to the Fermi energy [1], leading to a large discrepancy of the AHC in both the hole and electron pockets.

Supplementary References

[1] Z. Liang *et al.*, *Phys. Rev. X* **11**, 031026 (2021).

[2] F. Yu, T. Wu, Z. Wang, B. Lei, W. Zhuo, J. Ying, and X. Chen, *Phys. Rev. B* **104**, L041103 (2021).

[3] B. Song *et al.*, arXiv preprint arXiv:2105.09248 (2021).

[4] T. Wang, A. Yu, H. Zhang, Y. Liu, W. Li, W. Peng, Z. Di, D. Jiang, and G. Mu, arXiv preprint arXiv:2105.07732 (2021).

[5] Y. Song, T. Ying, X. Chen, X. Han, X. Wu, A. P. Schnyder, Y. Huang, J.-G. Guo, and X. Chen, *Phys. Rev. Lett.* **127**, 237001 (2021).

[6] Q. Yin, Z. Tu, C. Gong, Y. Fu, S. Yan, and H. Lei, *Chin. Phys. Lett.* **38**, 037403 (2021).

[7] E. Liu, Y. Sun *et al.*, *Nat. Phys.* **14**, 1125-1131 (2018).

[8] K. S. Takahashi, H. Ishizuka *et al*., *Sci. Adv.* **4**: eaar7880 (2018).

[9] L. Ye, M. Kang, J. Liu, F. v. Cube *et al*., *Nature* **555**, 638-642 (2018).

[10] Y. Shiomi, Y. Onose, Y. Tokura, *Phys. Rev.B* **79**, 100404(R) (2009).

[11] Y. Tian, L. Ye, X. F. Jin, *Phys. Rev. Lett.* **103**, 087206 (2009).

[12] L. Ye, Y, Tian, X. F. Jin, D. Xiao, *Phys. Rev. B* **85**, 220403(R) (2012).

[13] T. Suzuki, R. Chisnell, A. Devarakonda *et al.*, *Nat. Phys.* **12**, 1119-1123 (2016).

[14] S.-Y. Yang *et al.*, Sci. Adv. **6**, eabb6003 (2020).

[15] S. Iguchi, N. Hanasaki, Y. Tokura, *Phys. Rev. Lett.* **99**, 077202 (2007).

[16] N. Nagaosa, J. Sinova, S. Onoda, A. H. MacDonald, and N. P. Ong, *Rev. Mod. Phys.* **82**, 1539 (2010).

[17] S. Onoda, N. Sugimoto, and N. Nagaosa, *Phys. Rev. B* **77**, 165103 (2008).

[18] N. Sinitsyn, A. MacDonald, T. Jungwirth, V. Dugaev, and J. Sinova, *Phys. Rev. B* **75**, 045315 (2007).

[19] K. Nakayama, Y. Li, T. Kato, M. Liu, Z. Wang, T. Takahashi, Y. Yao, and T. Sato, *Phys. Rev. B* **104**, L161112 (2021).

[20] H. LaBollita and A. S. Botana, Phys. Rev. B **104**, 205129 (2021).

[21] K. Kobayashi and K. Nomura, Journal of the *J. Phys. Soc. Jpn.* **91**, 013703 (2022).

[22] G. Kresse and J. Furthmüller, *Phys. Rev. B* **54**, 11169 (1996).

[23] G. Kresse and D. Joubert, *Phys. Rev. B* **59**, 1758 (1999).

[24] J. P. Perdew, K. Burke, and M. Ernzerhof, *Phys. Rev. Lett.* **77**, 3865 (1996).

[25] A. A. Mostofi, J. R. Yates, Y.-S. Lee, I. Souza, D. Vanderbilt, and N. Marzari, *Comput. Phys. Commun.* **178**, 685 (2008).

[26] E. M. Kenney, B. R. Ortiz, C. Wang, S. D. Wilson, and M. J. Graf, *J. Phys. Condens. Matter* **33**, 235801 (2021).

[27] Y. Hu *et al.*, arXiv preprint arXiv:2106.05922 (2021).

[28] C. Wang, S. Liu, H. Jeon, and J.-H. Cho, *Phys. Rev. B* **105**, 045135 (2022).

[29] J. Zhao, W. Wu, Y. Wang, and S. A. Yang, *Phys. Rev. B* **103**, L241117 (2021).

[30] J.-F. Zhang, K. Liu, and Z.-Y. Lu, *Phys. Rev. B* **104**, 195130 (2021).
